# Supplementary material for: Molecular Modeling of Epithiospecifier and Nitrile-Specifier Proteins of Broccoli and Their Interaction with Aglycones
Source: Molecules. 2020 Feb 11;25(4):772. doi: 10.3390/molecules25040772 (PMC7071048; doi:10.3390/molecules25040772)
Supplement: Supplementary file 1 [file molecules-25-00772-s001.zip › Supplementary Tables.docx]

**Table S1.** Aminoacidic consensus sequence of NSP from broccoli homolog organisms (Q9SDM9, M4FIJ4, A0A078G479 and A0A0D3AD54)

| MAQKLEAKGGKKGGVWDDGVHDGIRKIYVGQGQDCIAFVKFEYADGSEVVVGDEHGKKTLLGTEEFEIDADDYLIYVEAFRDKATEETIVDLKFETYKGKTNKHIETSPGVKFVLHGGKIIGFHGRSSDVLHSLGAYVTFPSAPELQGKWIKVEQKGQAPGLRCSHAIAQVGNKIYAFGGELIPNQPIDKDLYVFDLETRTWSISPATGDVPHLSCLGVRMVSIGSTLYVFGGRDASRKYNGFYSFDTIKNEWKLLTPVEEGPIPRSFHSMAANENNVYVFGGVSATERLKNLDAYNIVDQKWVQCATPGESVSIRGGAGLEVVQGKVWVVYGFNGCEVDDVHYYDPVEDKWTQVETFGEKPSARSVFASAVVGKHIVIFGGEVAMDPQAHVGPGQLIGGTFALDTETLKWERLDKLGEDEETPEIRGWSASTSGTIDGKKGLVMHGGKAQTNGRFDDLFFYGTDSA |
| --- |

**Table S2.** Glucosinolates described in the literature.

| Compound | Formula | Mass (g/mol) | [M-H]-(m/z) |
| --- | --- | --- | --- |
| Diglucothiobeinin | C17H31N1O14S4 | 601 | 600 |
| 3M-hydroxy-glucoisatisin | C21H26N2O13S2 | 578 | 577 |
| Glucoisatisin | C21H26N2O12S2 | 562 | 561 |
| 9-methyl-sulfonylnonyl-GLS | C17H33N1O11S3 | 523 | 522 |
| 6-methyl-sulfonyl-3-oxo-hexyl-GLS | C14H25N1O12S3 | 495 | 494 |
| 4-methoxy-glucobrassicin | C17H22N2O10S2 | 478 | 477 |
| Neoglucobrassicin | C17H22N2O10S2 | 478 | 477 |
| 5-hydroxy-glucobrassicin | C16H20N2O10S2 | 464 | 463 |
| 4-hydroxy-glucobrassicin | C16H19N2O10S2 | 463 | 462 |
| Glucoerysolin | C12H23N1O11S3 | 453 | 452 |
| Glucoalyssin/glucoallysin | C13H25N1O10S3 | 451 | 450 |
| Glucobrassicin | C16H19N2O9S2 | 447 | 446 |
| Glucocheirolin | C11H21N1O11S3 | 439 | 438 |
| Glucosibarin | C15H21N1O10S2 | 439 | 438 |
| Glucoraphanin | C12H23N1O10S3 | 437 | 436 |
| Glucoraphenin | C12H21N1O10S3 | 435 | 434 |
| Trisulfanyl-glucocapparin | C8H14N1O9S5 | 428 | 427 |
| Glucosinalbin/Sibalbin | C14H19N1O10S2 | 425 | 424 |
| Glucoiberin | C11H21N1O10S3 | 423 | 422 |
| Gluconasturtin | C15H21N1O9S2 | 423 | 422 |
| Glucoerucin | C12H23N1O9S3 | 421 | 420 |
| Glucoraphasatin | C12H21N1O9S3 | 419 | 418 |
| Glucotropaeolin | C14H19N1O9S2 | 409 | 408 |
| Glucoiberverin | C11H21N1O9S3 | 407 | 406 |
| Glucosatavin | C11H21N1O9S3 | 407 | 406 |
| Glycinyl-glucocapparin | C10H17N2O11S2 | 405 | 404 |
| 3-methylpentyl-GLS | C13H25N1O9S2 | 403 | 402 |
| 4-methylpentyl-GLS | C13H25N1O9S2 | 403 | 402 |
| Gluconapoleiferin | C12H21N1O10S2 | 403 | 402 |
| Glucopoleiferin | C12H21N1O10S2 | 403 | 402 |
| Glucoraphasativusain | C13H25N1O9S2 | 403 | 402 |
| Disulfanyl-glucocapparin | C8H14N1O9S4 | 396 | 395 |
| Glucosisaustricin | C11H21N1O10S2 | 391 | 390 |
| 3-methylbutyl-GLS | C12H23N1O9S2 | 389 | 388 |
| Glucojiaputin | C12H23N1O9S2 | 389 | 388 |
| Glucokohlrabiin | C12H23N1O9S2 | 389 | 388 |
| Progoitrin/Epiprogoitrin | C11H19N1O10S2 | 389 | 388 |
| Glucobrassicanapin | C12H21N1O9S2 | 387 | 386 |
| Glucosisymbrin | C10H19N1O10S2 | 377 | 376 |
| Dihydrogluconapin | C11H21N1O9S2 | 375 | 374 |
| Glucocochlearin | C11H21N1O9S2 | 375 | 374 |
| Glucoconringianin | C11H21N1O9S2 | 375 | 374 |
| Gluconapin | C11H18N1O9S2 | 372 | 371 |
| Mercapto-glucocapparin | C8H14N1O9S3 | 364 | 363 |
| Glucoputrajivin | C10H19N1O9S2 | 361 | 360 |
| Isopropyl/n-propyl-GLS | C10H18N1O9S2 | 360 | 359 |
| Sinigrin | C10H17N1O9S2 | 359 | 358 |
| Glucocapparin | C8H14N1O9S2 | 332 | 331 |

**Table S3.** m/z signals and characteristic fragments of glucosinolates.

| Signal | Observed structure | Lost structure |
| --- | --- | --- |
| m/z 275 | C6H11O5-SSO3 |  |
| m/z 259 | C6H11O5-OSO3 |  |
| m/z 241 | C6H9O4-OSO3 |  |
| m/z 227 | C6H11O5-SO2 |  |
| m/z 195 | C6H11O5-S- |  |
| m/z 97 | HSO4 |  |
| m/z 80 | SO3 |  |
| [M-242-H]- |  | C6H10O5-SO3 |
| [M-196-H]- |  | C6H10O5-H2S |
| [M-178-H]- |  | C6H10O5-OH |
| [M-162-H]- |  | C6H10O5 |
| [M-113-H]- |  | SO4-OH |
| [M-97-H]- |  | HSO4 |
| [M-80-H]- |  | SO3 |
