# Supplementary figures and images for: Molecular Modeling of Epithiospecifier and Nitrile-Specifier Proteins of Broccoli and Their Interaction with Aglycones
Source: Molecules. 2020 Feb 11;25(4):772. doi: 10.3390/molecules25040772 (PMC7071048; doi:10.3390/molecules25040772)

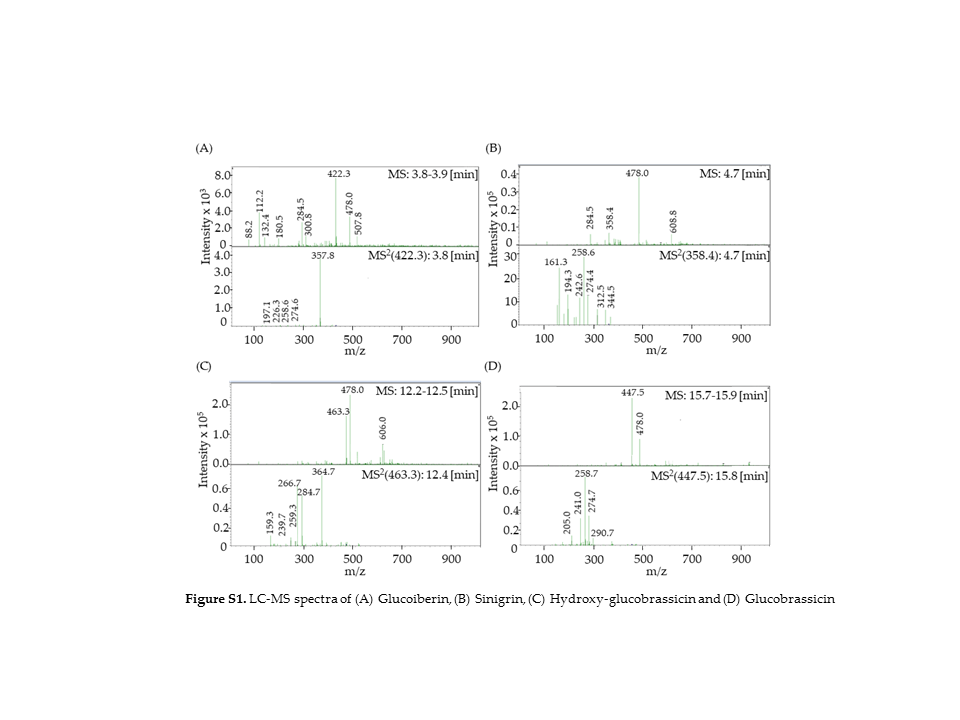

Supplement: Supplementary file 1 [file molecules-25-00772-s001.zip › Fig. S.1.tif]

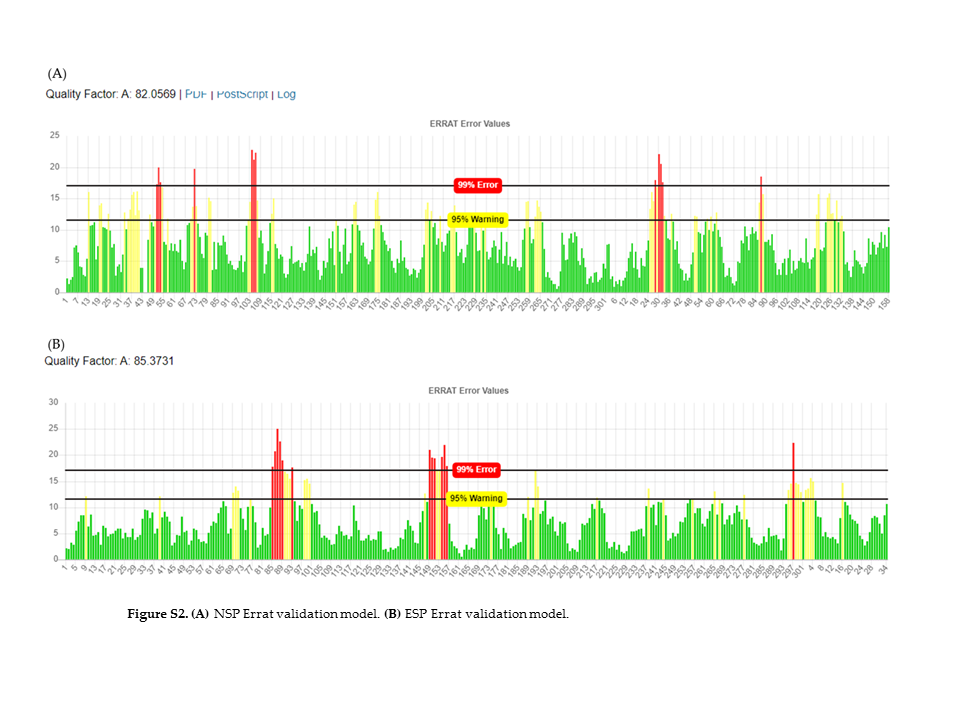

Supplement: Supplementary file 1 [file molecules-25-00772-s001.zip › Fig. S.2..tif]

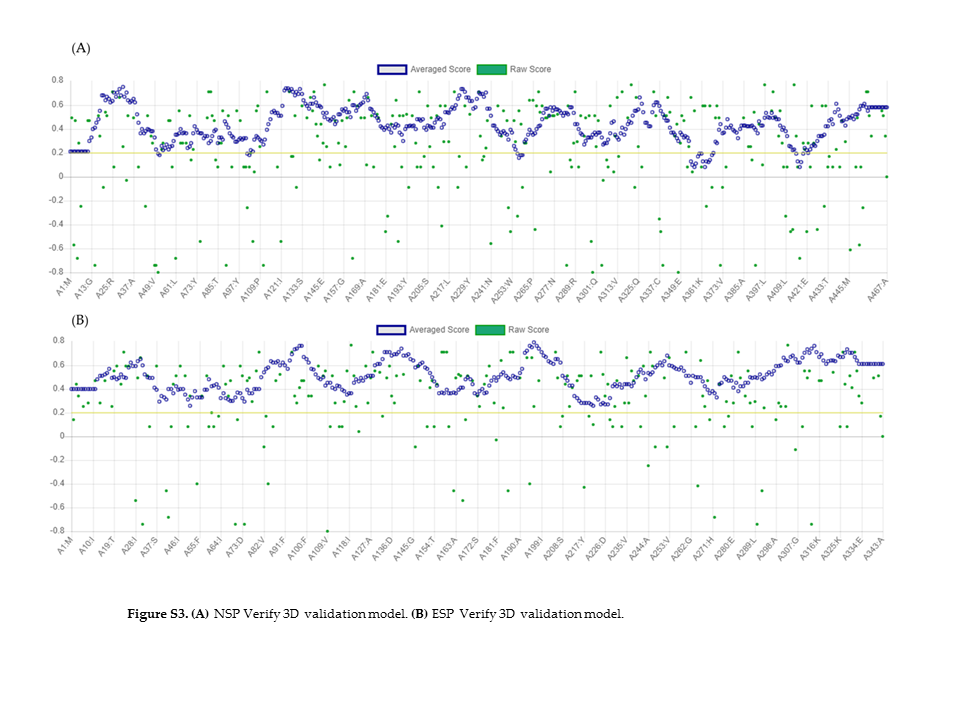

Supplement: Supplementary file 1 [file molecules-25-00772-s001.zip › Fig. S.3..tif]

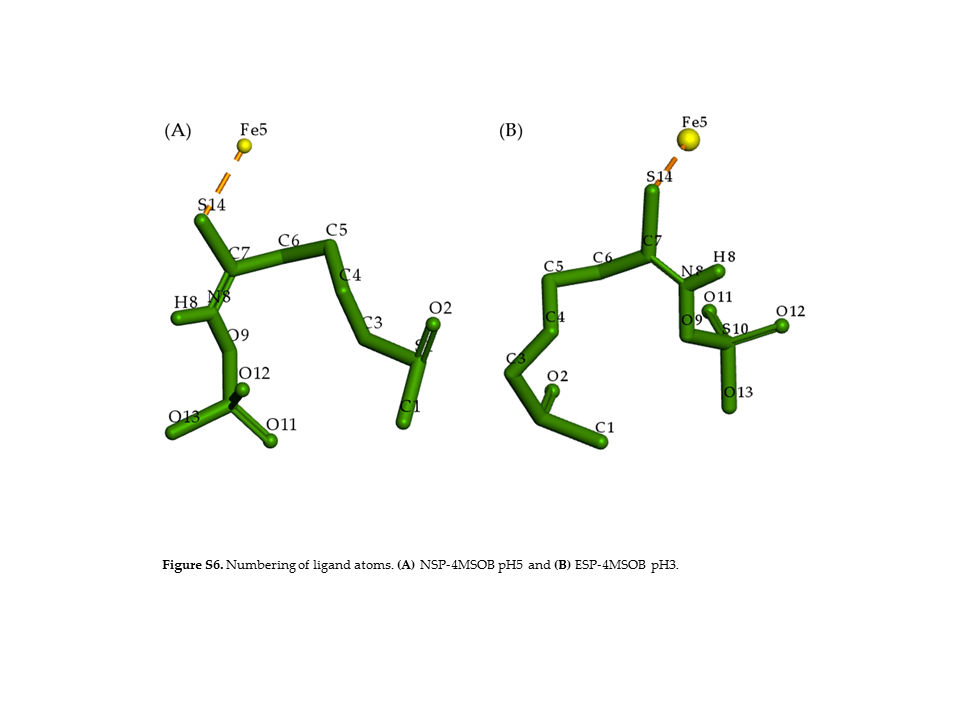

Supplement: Supplementary file 1 [file molecules-25-00772-s001.zip › Fig S.6.tif]

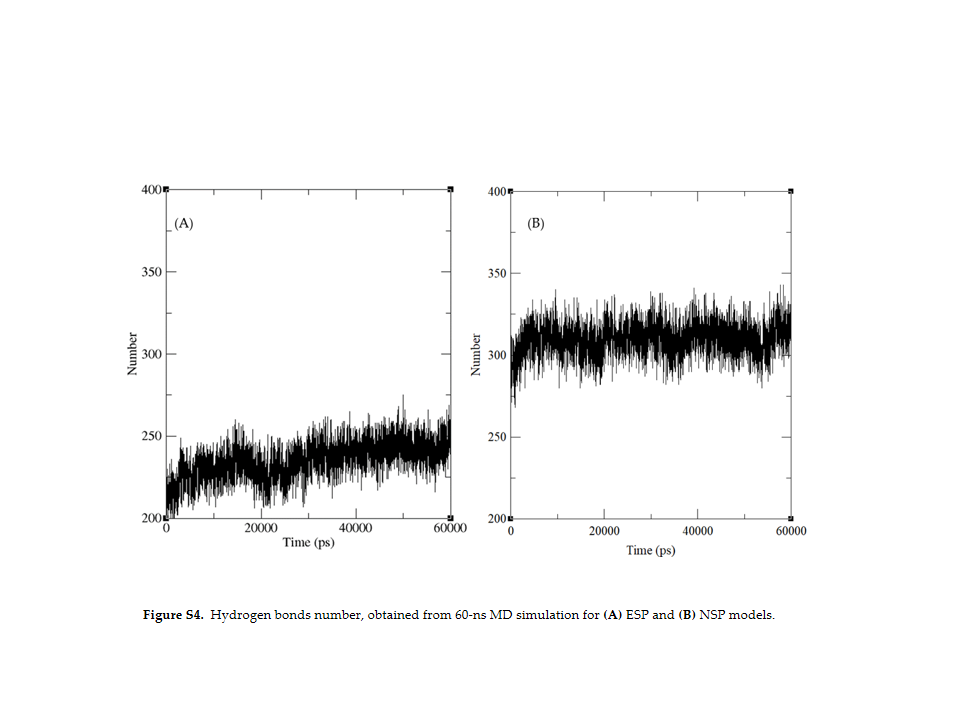

Supplement: Supplementary file 1 [file molecules-25-00772-s001.zip › Fig. S.4 rev.tif]

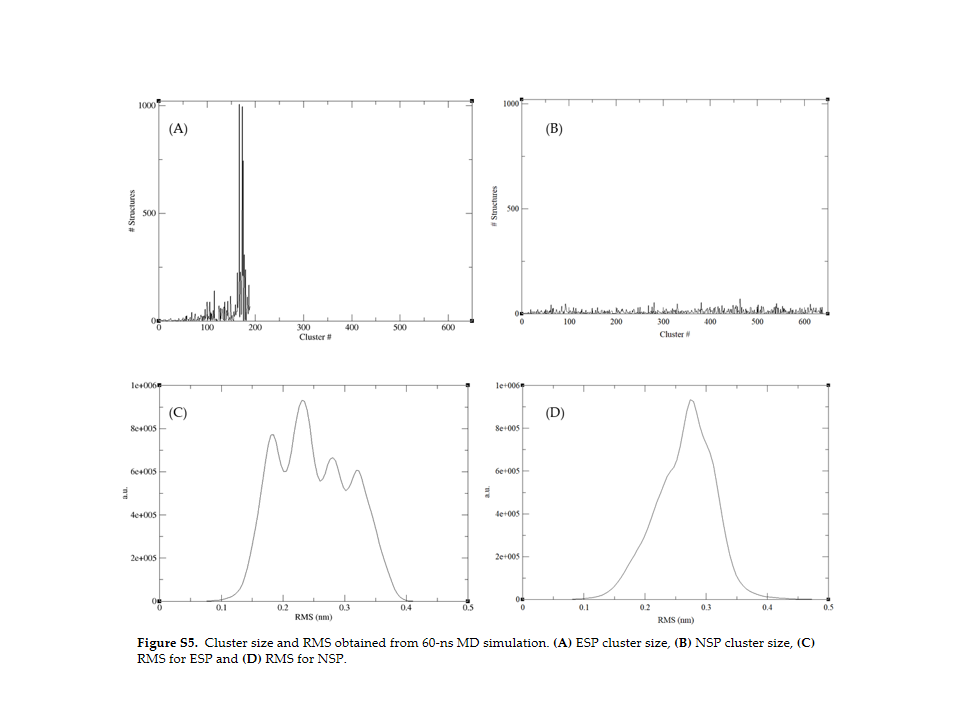

Supplement: Supplementary file 1 [file molecules-25-00772-s001.zip › Fig. S.5 rev.tif]
